# The impact of defects type and location on the integrity test results of RC piles in sand

**DOI:** 10.1038/s41598-026-67109-5

**Published:** 2026-08-25

**Authors:** Reham M. Samaan, Mohamed S. A. Saafan, Abdelsalam A. Mokhtar, Ahmed M. Ebid

**Affiliations:** 1 Faculty of Engineering, Ain Shams University, Cairo, Egypt; 2 Faculty of Engineering and Technology, Future University in Egypt, New Cairo, Egypt

**Keywords:** Pile Integrity Testing, Soil-Structure interaction, Defected Piles, Reflectogram, Engineering, Materials science

## Abstract

This research investigates the impact of pile defects on the prediction accuracy of pile toe during the low-strain integrity testing (LSIT). It assesses the conditions of concrete piles, especially piles with defects that are often difficult to identify through traditional investigation. The developed 3D FEM model in ABAQUS 2024 is validated using field data from projects in Egypt. The methodology includes this developing model and a parametric study of 90 cases, including intact and defective piles. The considered parameters are defect type, defect location, pile slenderness ratio and relative density of sand. The proposed model studied the effect of these parameters on the detection of the pile toe. It also investigates their effects on the reflectogram magnitude at the pile toe and at the defect location. This research describes the waveform response to the studied parameters and presents the effects of defects on LSIT results for piles. The FEM results showed that wave behavior depends on pile geometry, soil stiffness, defect location, and defect type. Defect type and location along the pile affect the pile toe detection in terms of the arrival time. An approximate linear relationship (R^2^ = 0.9983) is observed between the normalized defect response and the defect location along the pile. This indicates a nearly 53% reduction, as the defect is located near the pile toe. The proposed equation provides a practical method for estimating defect severity and its size as a function of relative defect magnitude and defect location in the pile.

## Introduction

Non-destructive testing (NDT) is an essential technology in civil engineering, especially for evaluating the structural integrity of pile foundations. NDT methods provide a reliable and efficient means of assessing the safety of a structural element. The continuous advancements of signal processing techniques have improved the accuracy and reliability of these assessments. These techniques are based on many physical theories. NDTs are used to detect anomalies in structural elements, such as defects, stresses, and microstructural degradation^1,2^. NDT involves testing, evaluation, and realistic assessment without damaging the structural component. To prevent damage and loss of structural integrity, early detection is essential. Also, one of the key objectives of NDT is determining defect type, location, size, shape, and orientation. Its evaluation involves assessing the defect and its impact on the structural element’s performance. NDT techniques are classified as static and dynamic. For real-time monitoring during service, dynamic techniques are used, and static techniques are used for offline inspections^3^. Several factors are considered when selecting the NDT technique, including inspection task standards and defect location^4–6^. Different NDT methods are suitable for various materials^7^. Environmental factors such as temperature, humidity, and soil conditions could affect the accuracy of NDT results^8,9^. Concrete piles can suffer defects and degradations such as voids, necking, cracks, concrete segregation, and reinforcement corrosion, which affect their structural safety. Most pile defects are visually inaccessible, but NDT and structural health monitoring (SHM) methods enable assessment of pile integrity^10^. Low-strain integrity testing (LSIT) is well-established as a quality-assurance method for concrete piles. It is a low-cost technique for anomaly detection or pile depth detection. A low-strain impulse is applied to the pile head using a handheld hammer. An accelerometer monitors the wave from the pile top. The impulse produces a stress wave that travels through the pile. This wave reflects changes in pile characteristics^11,12^. LSIT is widely applied in quality assurance for deep foundations, including bored and driven piles. LSIT analysis depends on the linear one-dimensional wave equation. The travel direction of stress waves is identical to the direction of the particles’ motions in compression waves. Common techniques include impact echo and cross-hole sonic logging. This technique verifies the detection of the pile toe, continuity, and concrete quality by interpreting the wave propagation behavior and its reflections^13^. The standard procedure for assessing pile quality using LSIT is a hammer^14^. This pile head generates low-energy transient vibrations within the elastic region of the pile material. The vibrations, generated without damaging the piles, enable assessment of reflected wave signals for pile integrity. The signals are measured by sensitive sensors such as accelerometers or geophones at the head of the pile. This sensor provides a velocity-time or acceleration signal to detect anomalies and determine the integrity of the pile^15^. The LSIT detects the defects in the pile, such as cracks, necking, or voids. In addition to pile length, ASTM D5882, EN 1997-1 (Eurocode 7), and BS EN ISO 22477-4:2018 standardize the procedure of LSIT, including setup, data acquisition, data analysis, and interpretation of the reflectograms^16,17^. Multiple impact tests should be performed to improve data reliability and reduce noise. The pile surface must be well-prepared for this test and bonded to the sensors to record acceleration and velocity. The impact load from the handheld hammer should be near the motion sensors. The readings are recorded using an accelerometer or a geophone. It is also recommended to check multiple test points on the pile surface for piles with diameters greater than 500 mm. The pile could be tested after concrete reached 75% of its strength or at least 7 days after casting. These standards help to ensure reliability, integrity, and consistency of the test. The test results show variations in pile geometry, cross-sectional area, and material properties. Pile toe depth is determined by analyzing reflectograms, wave velocities, pile length detection, and amplification factors. The structural evaluation interprets impedance changes in wave propagation within the pile by analyzing signal reflections and comparing data from multiple tests conducted on the same pile. Based on collected data, defects, if detected, are quantified using computational methods. Generally, interpretation depends mainly on human experts, who integrate analysis, test records, loading conditions, and soil properties^16^.

Wave propagation theory is the basis for interpreting the dynamic behavior of piles subjected to impact loads. Compressive wave that will move downward through the pile at a speed (* C*) determined by Eq. (1). The material’s density is indicated as ρ, and E represents Young’s modulus.

1 $$\:\mathrm{C}=\sqrt{\mathrm{E}/{\uprho\:}}$$

In simpler terms, the 1D longitudinal wave propagation is described in Eq. (2). Also, Eq. (3) represents the second-order partial differential equation for 3D wave propagation.

2 $$\:\frac{{\partial\:}^{2}u}{\partial\:{t}^{2}}=\:{c}^{2}\frac{{\partial\:}^{2}u}{\partial\:{x}^{2}}$$

3 $$\:\frac{{\partial\:}^{2}u}{\partial\:{t}^{2}}=\:{c}^{2}[\frac{{\partial\:}^{2}u}{\partial\:{x}^{2}}+\frac{{\partial\:}^{2}u}{\partial\:{y}^{2}}+\:\frac{{\partial\:}^{2}u}{\partial\:{z}^{2}}\:]\:$$

Where c is the wave velocity, u represents the wave’s displacement, t is time, and x, y, and z represent spatial coordinates^18^. Equation (4) describes the pile impedance Z, where A is the pile’s cross-sectional area. The motion of the wave depends on the relation between the hammer’s impedance and the pile’s impedance^19^. The impact force is related to the velocity ν through the pile impedance expressed as F = Z·ν.

4 $$\:Z=\rho\:\cdot\:A\cdot\:C$$

The accuracy of defect detection and localization depends on the wave propagation models. To ensure the reliability of these models, certain factors should be considered, including the pile geometry, material properties, and soil-pile interaction effects^20^.

Finite Element Modelling (FEM) is used to model the dynamic response in the LSIT, which helps in understanding how waves propagate within the pile and the effect of defects on the measured signal. FEM compares the incident wave and the reflected wave to determine whether each reflection is from the pile tip or a defect along the pile. In addition, FEM enables precise and controlled study of sensor placement sensitivity, which is preferable to field tests given practical constraints and site conditions that affect reading accuracy^23^.

## Literature review

### Test results and wave propagation analysis

The FEM method has been widely used to evaluate integrity testing, detect defects, and analyze the pile-velocity response after impact loading at the pile head. Zheng et al. presented analytical and numerical models demonstrating that geometric and material defects significantly affect test signals. 3D models showed high-frequency vibrations that complicate signal interpretation, and the negative response after the first positive peak can be misinterpreted as a defect near the pile head, when it is a 3D effect. It is recommended to place the sensor at 2R/3 from the pile center and 0.5R from the impact point^24^. However, their model was applied to a single-pile geometry with a fixed defect location, without addressing the effects of soil density, pile slenderness ratio, or varied defect locations along the pile length on the velocity-time reflectograms. Masoumi et al. conducted LSIT on a single cast-in-place pile and a numerical model. The developed computational model predicted vibrations occurring in the soil around the pile. When comparing the model with the actual measurements, the model performed well in areas far from the pile (*r* > 4 m). In nearby areas, the model overestimated the measured values. However, their numerical model was conducted only for intact-pile scenarios with no defects^25^. FEM was used in tests of large-scale models to detect pile defects, and the efficiency of LSIT was compared with other testing methods, such as sonic logging. To improve detection accuracy for site-cast piles, different input pulse durations were evaluated. It was recommended to use hammers of varying weights and materials to improve data quality in field tests^26,27^. In the large-diameter pile, high-frequency interference complicates the interpretation of LSIT and makes it difficult to use the 1D model. A developed model shows that the peak response decreases in stiff soils and those with a low Poisson ratio, and that wider pulses reduce this interference. To improve the test’s reliability, it is recommended to use soft hammers and place sensors at 90-degree angles around the pile^28^. The deviation in the impact position causes high-frequency disturbances and leads to incorrect interpretation of pile integrity. The optimal position of sensors is determined to be between 0.5R and 0.7R from the center of the pile^29^. The interpretation of LSIT based on 1D stress-wave theory does not account for the complex wave patterns around the pile head. This condition occurs when the pulse wavelength exceeds 4 times the pile radius. For reliable interpretation, the sensor must be placed at least 0.5R from the pile center^30^. Ayasrah et al. used verified 3D FEM simulations to investigate the effects of friction angle, pile length, end-bearing capacity, and the lateral displacement responses of pipe piles. Their results show that sand stiffness and strength parameters have a significant influence on the 3D pile behaviour in loose sand. The numerical analysis of pile-soil interaction and sandy properties is important for demonstrating the performance of piles under complex loading conditions. Soil-structure interaction and sand stiffness were also found to have significant importance on the dynamic response of pile foundations. The base-ground accelerations, the foundation displacements, and the internal forces are affected by the properties of sandy soil and the pile-soil coupling under seismic loading^31,32^. Nazir and Hussien developed a theoretical model that accounts for soil resistance and damping in the one-dimensional wave equation for pile integrity testing and applied it to piles in dense sandy soils. Their results quantify the effect of soil damping in sand on the attenuation of stress-wave force and velocity along the pile, leading to a reduction in the amplitude of reflections at depth and to a different interpretation of the low-strain PIT signals in granular deposits^33^. Auersch studied wave propagation and foundation vibrations in layered soils, such as sand layers whose stiffness increases with depth. The study, using wavenumber-domain solutions, shows that thin, soft sand layers overlying stiffer sand significantly modify low-frequency damping and high-frequency attenuation, which in turn affect the vertical and horizontal vibration amplitudes at foundations, with direct relevance to low-strain pile vibration and LSIT signal behavior in layered sandy soils^34^.

This study aims to fulfill a research gap, which is the impact of pile defects on the pile toe prediction accuracy by simulating the low-strain pile integrity test using a FEM model. The main objective is to evaluate the impact of pile slenderness ratio (L/D), defect location, and defect type in different soil types on the error in the toe location and on the magnitude of the reflectogram at the defect location.

### Methodology

**Fig. 1:**
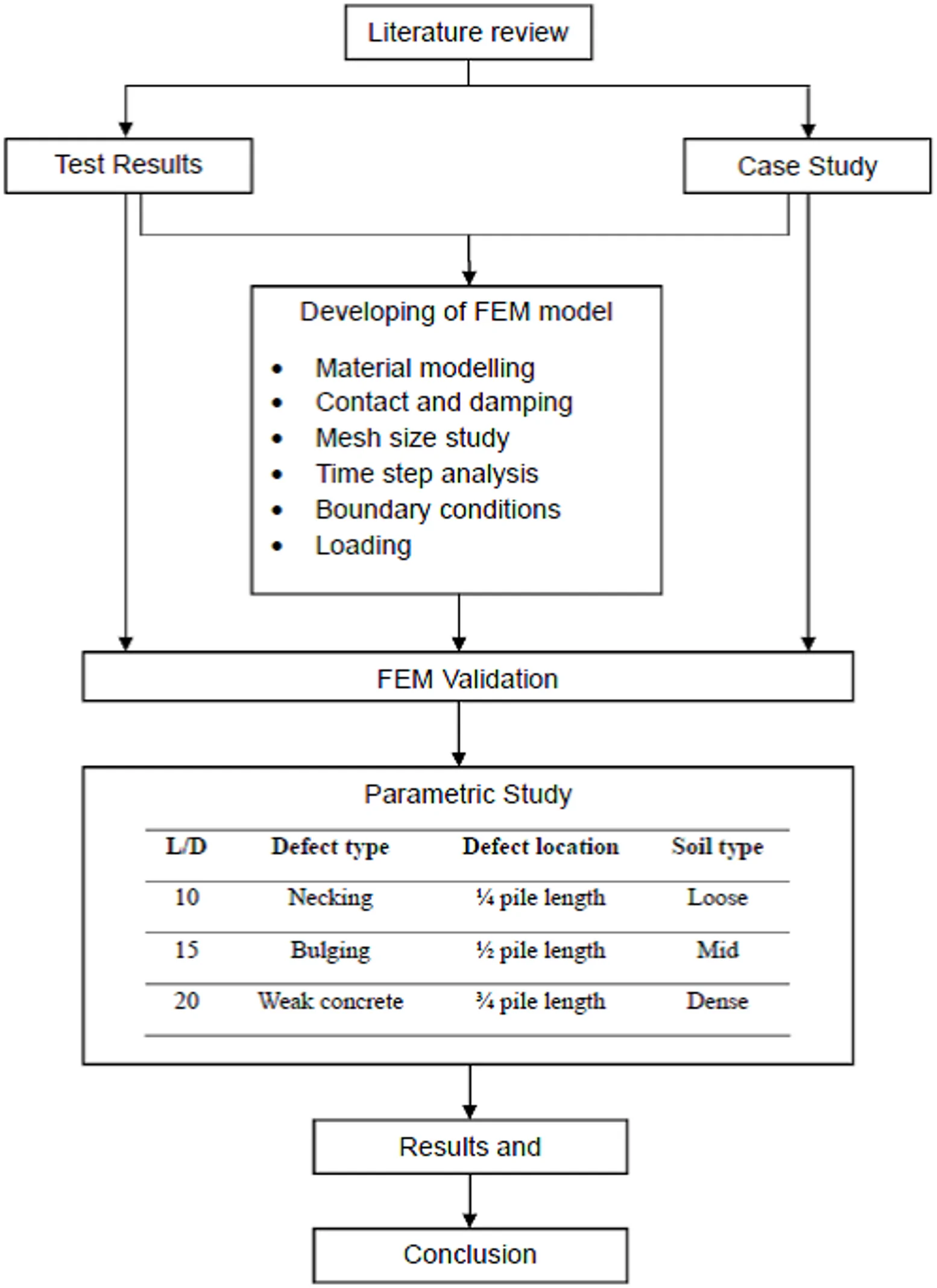
Methodology framework.

The dynamic response of defective piles subjected to Low-Strain Integrity Testing (LSIT) was analyzed using a developed 3D Finite Element Method (FEM) framework. The FEM focuses on simulating the wave behavior propagation in piles with defects such as necking, weak concrete zones, and bulging. The resulting velocity-time reflectogram is analyzed for variations in defect position, defect type, and surrounding soil conditions. Previous studies indicated that the reliability of small-amplitude signals is constrained by experimental conditions, such as sensor sensitivity, environmental noise, and field limitations, which could not capture the effects of defects on the reflectogram. In contrast, the 3D FEM approach allows for high-resolution simulations of wave propagation and the resulting reflections. The finite element simulations assess the conditions of the defects and their impact on the detection of the pile toe and the relative magnitude reflectogram at the pile toe and the defect. These FEM simulations were conducted using ABAQUS 2024 software. The methodology used in this research follows the FEM framework for LSIT-related wave propagation, as illustrated in Fig. 1.

### Material modelling

The concrete and sand parameters used in the finite element simulations were determined based on a study by Ebid, A^33^. Three sand types were modeled, each with different unit weights, stiffnesses, and damping ratios. According to the Canadian Foundation Manual, appropriate damping values selected for the sandy soil were 3%, 4%, and 5%^34^. Table 1 summarizes the material properties considered for the parametric study.

**Table 1:** Considered materials properties for parametric study.

Material	Unit weight (kN/m^3^)	Young’s modulus (MPa)	Poisson’s ratio	Damping ratio (%)	Rayleigh Damping coefficients
Concrete	24	38,400	0.2	2	α = 17.45 β = 1.27 × 10^− 5^
Sand 1 (Loose)	13	15	0.3	5	α = 0.363 β = 0.00382
Sand 2 (Medium)	16	115	0.3	4	α = 0.725 β = 0.00123
Sand 3 (Dense)	18	400	0.3	3	α = 0.957 β = 0.000523

### Contact and damping

The contact interface between the pile and the soil was defined in Abaqus using both friction and frictionless formulations. This interface was modeled with a default hard contact to reflect the expected behavior, allowing for separation after contact when necessary. A penalty friction formulation with an isotropic friction coefficient characterized tangential behavior. Damping ratios for each material were expressed as Rayleigh coefficients for ABAQUS. The conversion relationship for Rayleigh damping is given by^35^.

5 $$\:\zeta\:\left(\omega\:\right)=\frac{\alpha\:}{2\omega\:}+\frac{\beta\:\omega\:}{2}$$

Where ζ(ω) is the target damping ratio at angular frequency ω, and α and β are the Rayleigh mass and stiffness proportional damping coefficients, respectively^36^.

6 $$\:{f}_{n}=\frac{(2n-1){V}_{s}}{4H},\:{V}_{s}=\sqrt{\frac{E}{2(1+\nu\:)\rho\:}}$$

Where Vs is the shear wave velocity, E is Young’s modulus, ν is Poisson’s ratio, ρ is the material density, and H is the height. The calculated α and β values were then input into ABAQUS to reproduce the desired damping behavior for each material type.

### Mesh size study

**Fig. 2:**
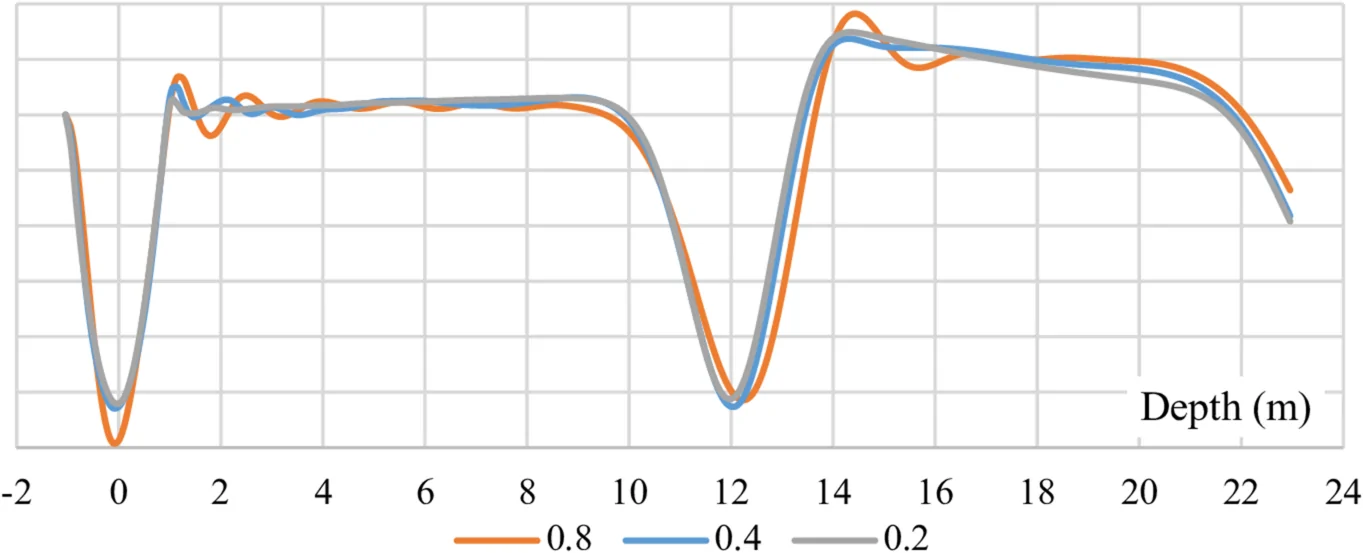
Mesh sensitivity analysis: velocity-time reflectograms for intact pile.

**Fig. 3:**
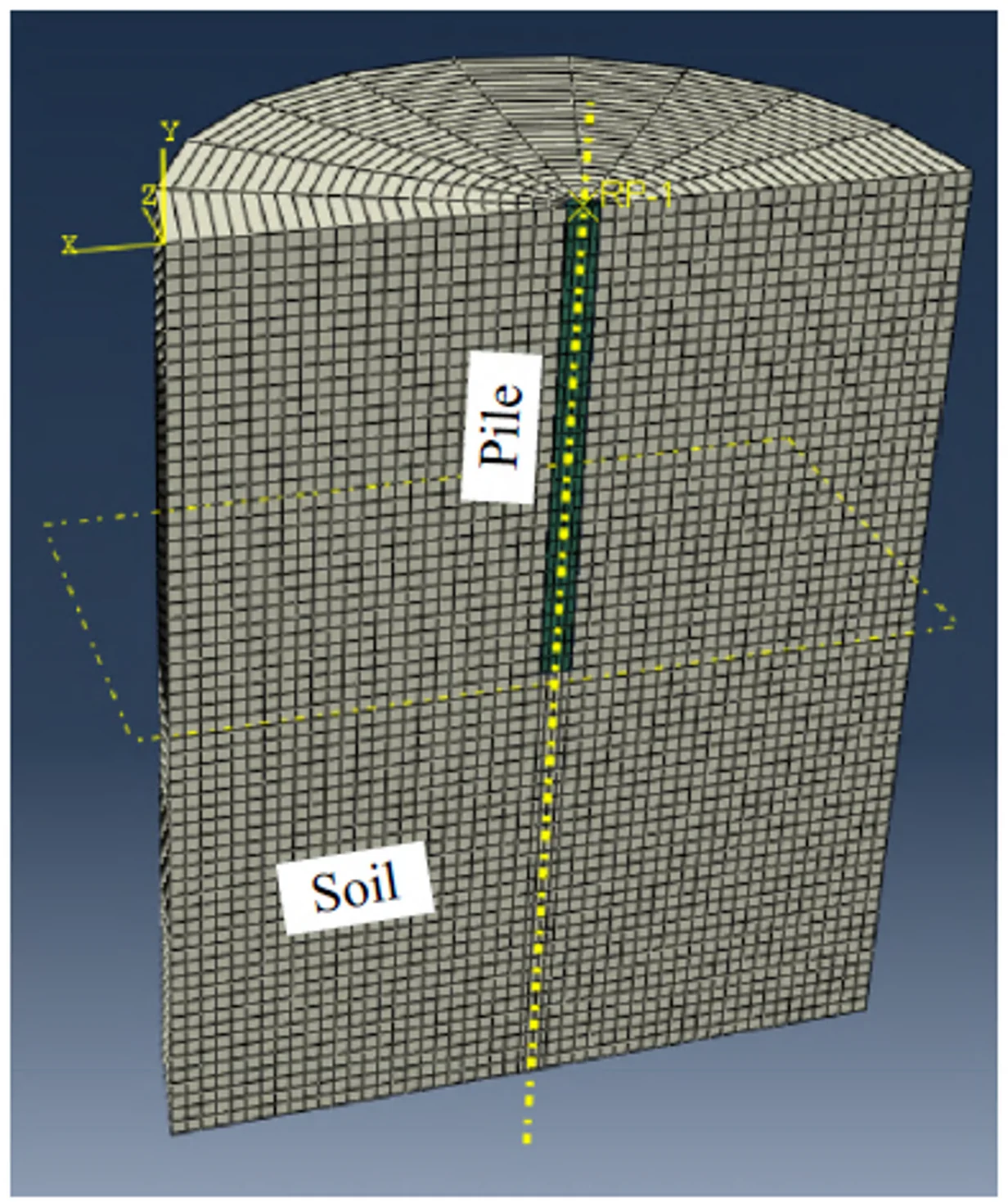
Mesh size.

An optimal element size was determined by a mesh sensitivity analysis to ensure accuracy and efficiency of the FEM. Three different mesh sizes, 0.2, 0.4, and 0.8, were analyzed for the intact pile model with the same loading and boundary conditions. Figure 2 illustrates the resulting velocity-time reflectograms compared for each mesh size. 0.2 and 0.4 mesh sizes showed nearly the same velocity-time reflectogram. However, the finer mesh size of 0.2 required more computational time, making it difficult to simulate multiple defect and soil-type models. On the other hand, the 0.8 mesh size indicates differences in the wave compared with the other two profiles and in the arrival time. Based on this mesh study, a uniform mesh size of 0.4 was selected for the pile-soil model, as it accurately represents wave propagation while keeping computational time reasonable, as shown in Fig. 3. The analysis utilized 8-node linear brick elements with reduced integration (C3D8R), enabling efficient and accurate wave propagation simulation in dynamic analyses.

### Time step analysis

The total analysis time was calculated based on the time required for stress waves to travel through the pile and then be reflected. This expression used the following equation, where L represents the length of the pile and * C* denotes the wave velocity in the pile material.:

7 $$\:T=\frac{2L}{C}$$

The wave velocity was approximately 4000 m/s based on the material parameters listed in Table 1. The maximum allowed time increment size (Δt) is calculated by dividing the total time T by 230 steps.

8 $$ \Delta {\mathrm{t}} = {\mathrm{T}}/230 $$

This analysis ensures a sufficient dynamic response to capture propagation behavior, wave reflections, and interference during simulation. This helps maintain numerical stability and computational efficiency in dynamic analysis.

### Boundary conditions

**Fig. 4:**
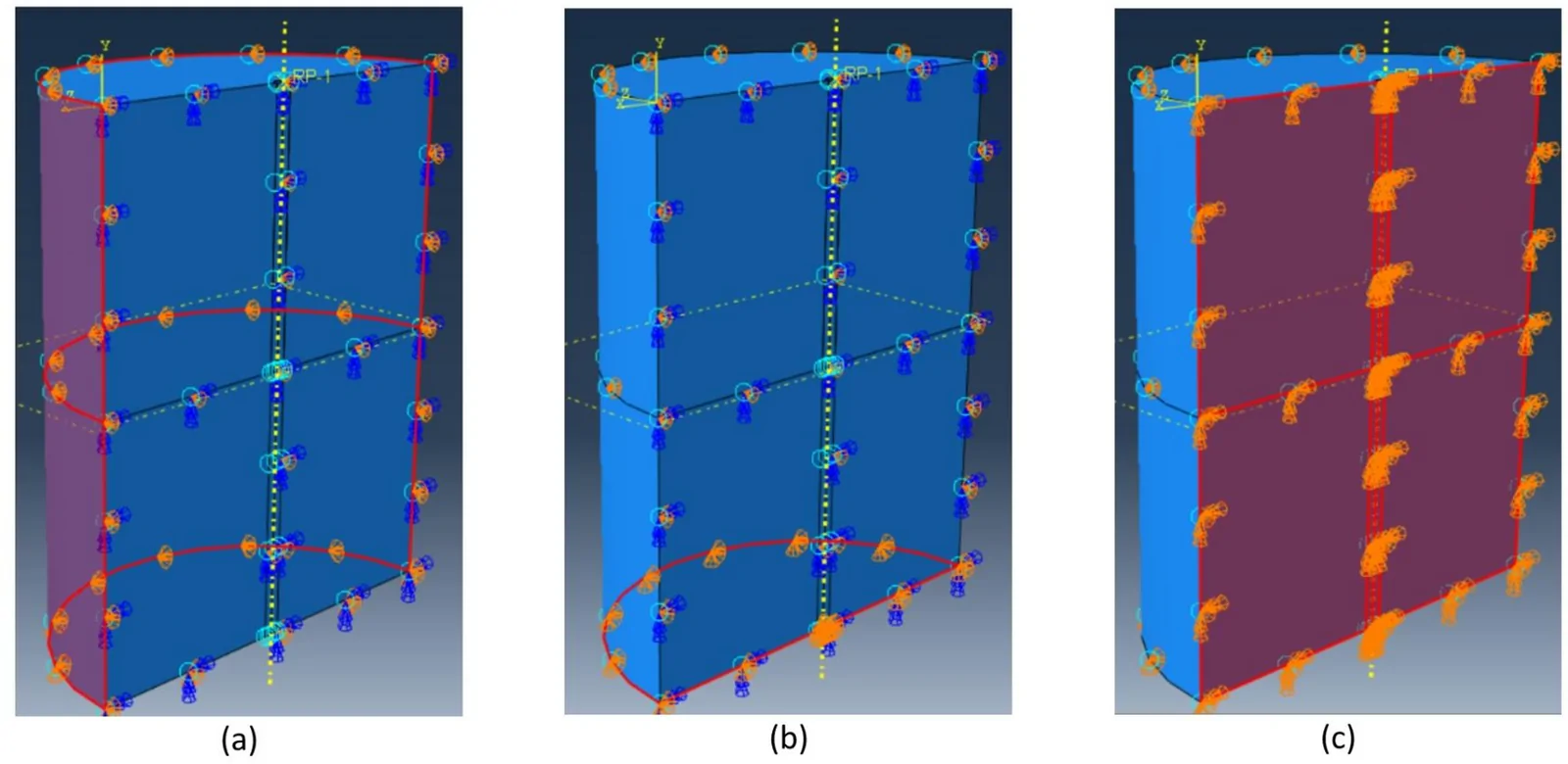
Boundary conditions (**a**) Outer skin condition (**b**) Bottom condition (**c**) Symmetric plane condition.

Three boundary conditions were applied in the FEM to simulate the pile-testing conditions. For the outer layer of the soil, where U1 = U3 = 0, the horizontal displacement is restricted, and the vertical displacement along the pile axis is allowed. The bottom layer simulates a rigid base condition and is constrained in all translational directions, with U1 = U2 = U3 = 0 to prevent any unrealistic displacement at the base of the model. For modeling only half of the model, the system is assumed to be symmetric about the Z-axis, with the ZSYMM boundary condition applied. These constraints, the vertical displacement U3 = 0, and U1 and U2 are permitted, as shown in Fig. 4.

### Loading

The impact load is applied to the pile surface for dynamic excitation using a 2.5 Kg handheld hammer, which aligns with the typical testing equipment in ASTM D5882^16^. Using appropriately timed light hammer pulses helps detect defects in the pile, whereas a very short pulse duration may cause misleading reflections in the signal that do not represent defects^21,22^. According to ASTM D5882, the impact duration for LSIT in the simulated model is approximately 1ms. The impulse sinusoidal equation was used to model the longitudinal transient excitation force P(t) at the pile head.

9 $$\:p\left(t\right)={P}_{0}\mathrm{sin}\left(\frac{\pi\:t}{T}\right)$$

**Fig. 5:**
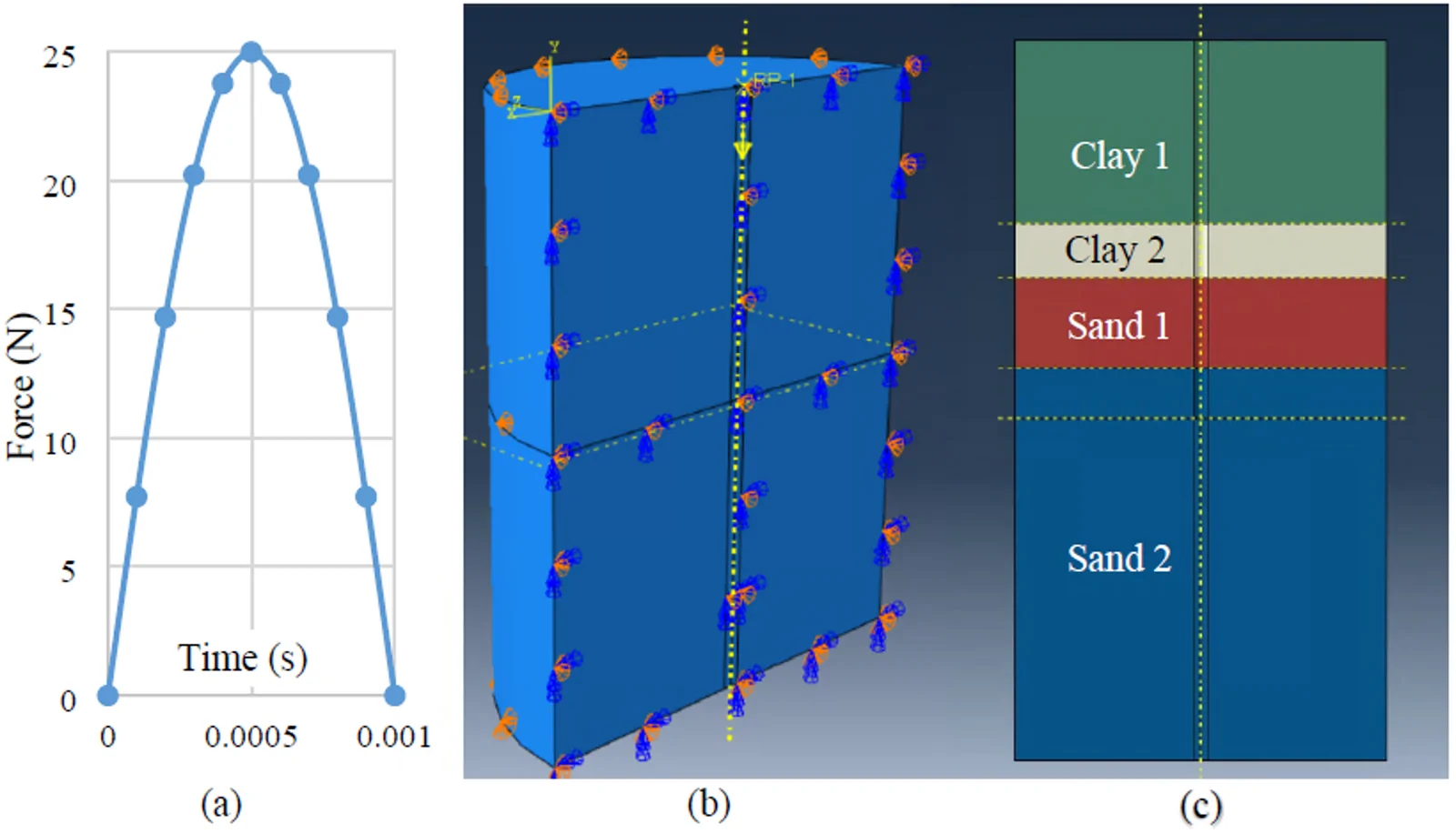
Verification model, (**a**) impact force pulse, (**b**) Loading condition, (**c**) Soil layer.

Where $\:{P}_{0}$ is the maximum transient excitation force of 25 N, t is the time, and T is the duration of the excitation force. Figure 5(a) shows a smooth half sine wave was created to reach its peak at the midpoint and returns to 0 at 1 ms as the force increases from 0 to a maximum value at 0.5 ms^37^. The impact load amplitude is represented as a tabular function in ABAQUS. Figure 5(b) shows the applied load of the hammer’s position at the center of the pile. As recommended for sensor locations at the pile head, the velocity-time reflectogram was analyzed at a radial distance of 0.5R, aligned with the sensor placement^38^. This reduces high-frequency interference and captures the wave propagation behavior more accurately.

### Validation of the finite element model

The LSIT data collection was obtained from construction projects across Egypt. The experimental reflectograms used for model validation were provided by the Nile Engineering Consulting Bureau (NCEB). The digitized velocity-time reflectograms from these field measurements were compared with the developed finite element model (FEM). This comparison ensures the reliability of the FEM in assessing pile integrity.

Validation was conducted on two groups of piles, each with a diameter of 0.8 m and lengths of 17 m and 24 m. The soil profile consists of multiple layers, as illustrated in Fig. 5(c). The upper soil layer, extending from 0 to 6 m, is classified as soft to medium silty clay. The second layer is sandy silty, followed by fine to medium sand down to a depth of 33 m. The wave velocity, C, was determined from the arrival times of each tested pile. For the 17 m piles, a wave velocity of 4100 m/s was recorded, while for the 24 m piles, a velocity of 4300 m/s was observed. These experimental reflectograms obtained from the LSIT are used to validate the finite element model (Table 2).

**Table 2:** Soil properties of validation case studies.

Material	Thickness (m)	Unit weight (kN/m³)	Young’s modulus (MPa)	Poisson’s ratio	Damping ratios
Pile1	17.0	24.0	40,344	0.2	2%
Pile2	24.0	24.0	44,376	0.2	2%
Clay1	10.2	16.0	2	0.45	5%
Clay2	3.0	16.0	3.5	0.45	4%
Sand1	5.0	20.0	550	0.3	3%
Sand2	21.8	22.0	660	0.3	3%

**Fig. 6:**
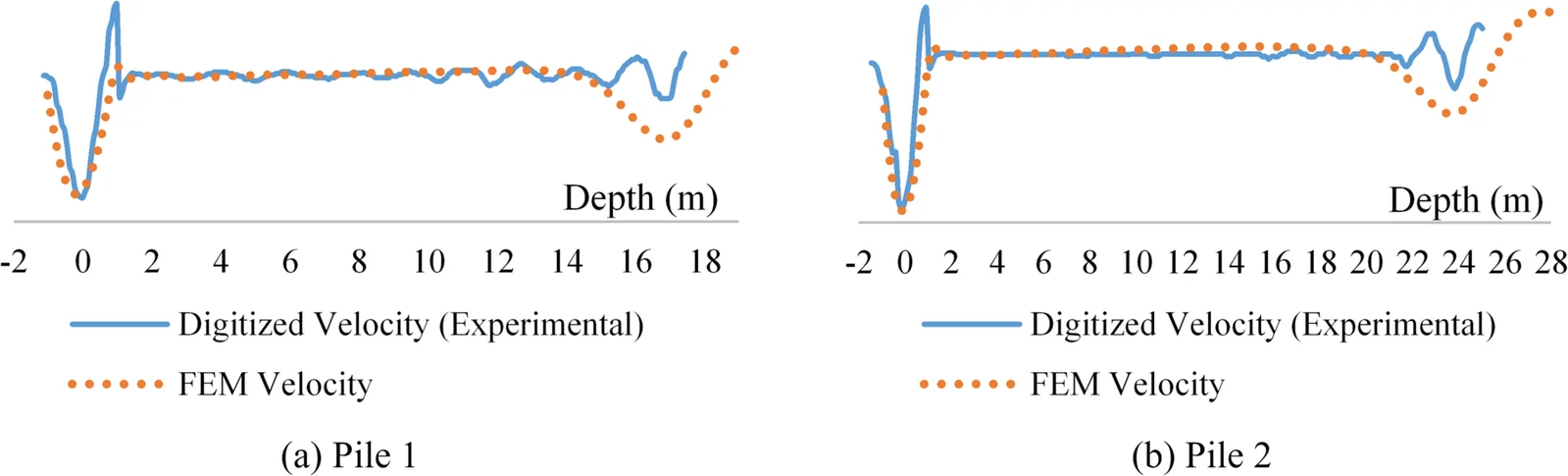
Comparison between digitized experimental data and finite element simulation results. (**a**) Pile 1, (**b**) Pile 2.

The corresponding experimental results and the resulting reflectograms were compared to ensure the reliability of the numerical simulations. The comparison focuses on accurately detecting the pile toe and on wave arrival times. Figure 6 demonstrates a strong correspondence between the digitized velocity and the simulated velocity-time reflectogram. It also illustrates the behavior of the wave propagation along the pile. Generally, this assessment helps develop a parametric study of defective piles and assesses the impact of the defects on LSIT results for the developed model.

### Parametric study

**Fig. 7:**
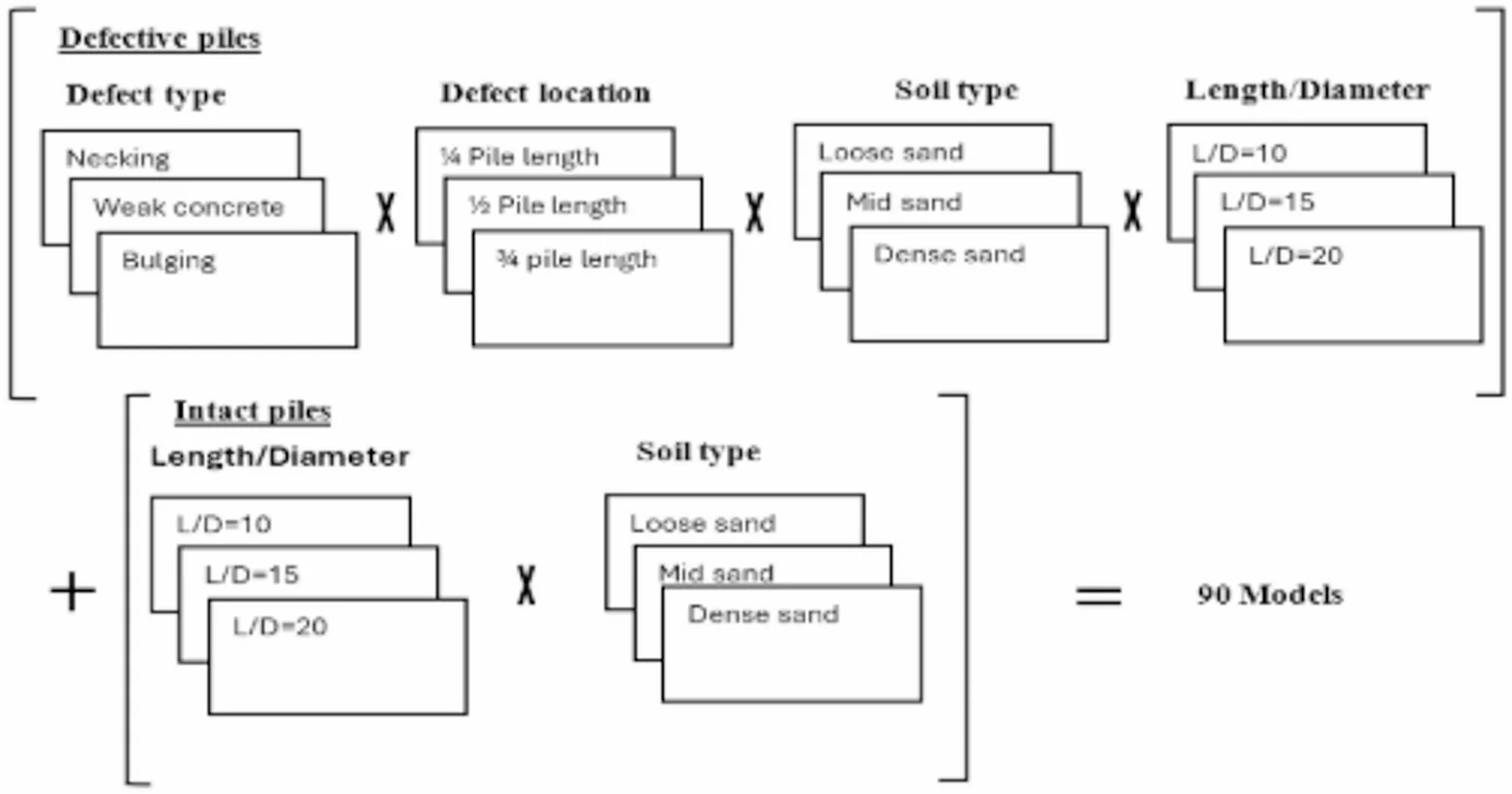
Parametric study.

**Fig. 8:**
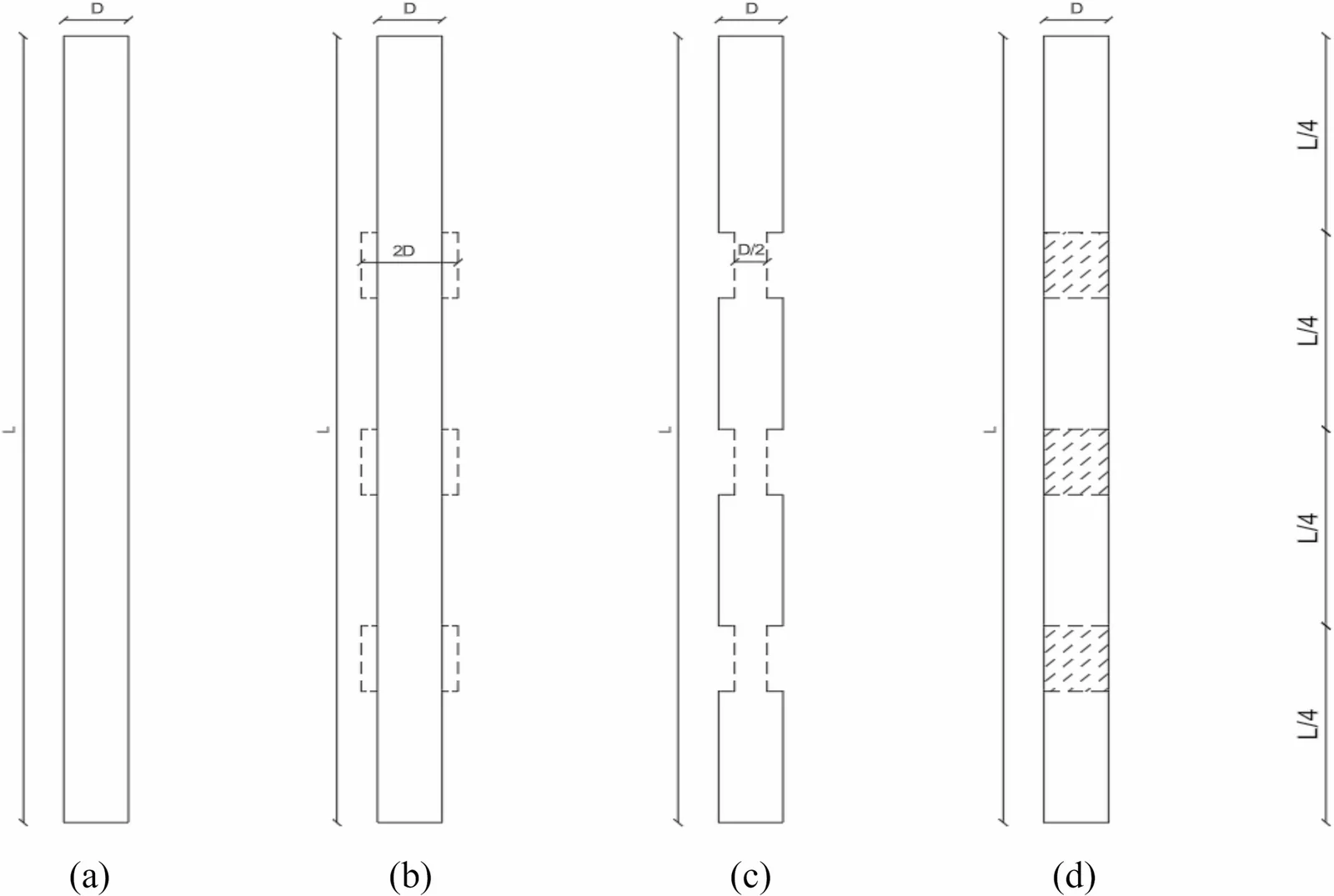
Pile models: (**a**) Intact pile (**b**) Bulging defect (**c**) Necking defect (**d**) Pile with weak concrete.

Figure 7 illustrates a developed parametric study on the impact of defects in RC piles through LSIT. The parametric study showed the effects of the defects on the resulting reflectograms and on wave propagation behavior. Both intact and defective piles are represented in 90 models. This study focused on parameters for the defective pile: defect type, defect location, soil type, and pile slenderness ratio (L/D). Regarding the intact piles, the L/D ratio and soil type are considered in the modeling, since no defect type or location is applied. For the defective pile models, all four parameters of defect type, defect location, pile slenderness ratio, and soil type are applied. Three types of defects were modeled: variations in pile diameter through necking, bulging, and weak concrete zones. Four conditions of pile models are represented, with the first one is an intact pile with a diameter (D) and a length (L) of 12 m. The second condition involves a localized increase in diameter of D/4 on each side (left and right) as a bulging defect. In the third case, the pile diameter decreases by D/2 due to a necking defect. The fourth one represents a weak concrete zone, with Young’s modulus reduced by half. All defect types were modeled with a fixed length of 1 m. It is located at three different locations along the pile: 25%, 50%, and 75% of the total length. This variation in defect position helps evaluate the effect on the reflected wave and on detection sensitivity. The variation in soil stiffness, as represented by three soil types: loose, medium, and dense sand, was defined to study their effects on the resulting reflectograms. Additionally, three different slenderness ratios of 20, 15, and 10, corresponding to (D = 0.6, 0.8, and 1.2 m), with a constant pile length of 12 m, are used to illustrate the impact on pile toe detection and the relative magnitude reflectogram. This parametric study enables evaluation of the impact of these parameter variations on wave propagation behavior and the resulting velocity-time reflectogram of the defective RC piles (Fig. 8).

**Fig. 9:**
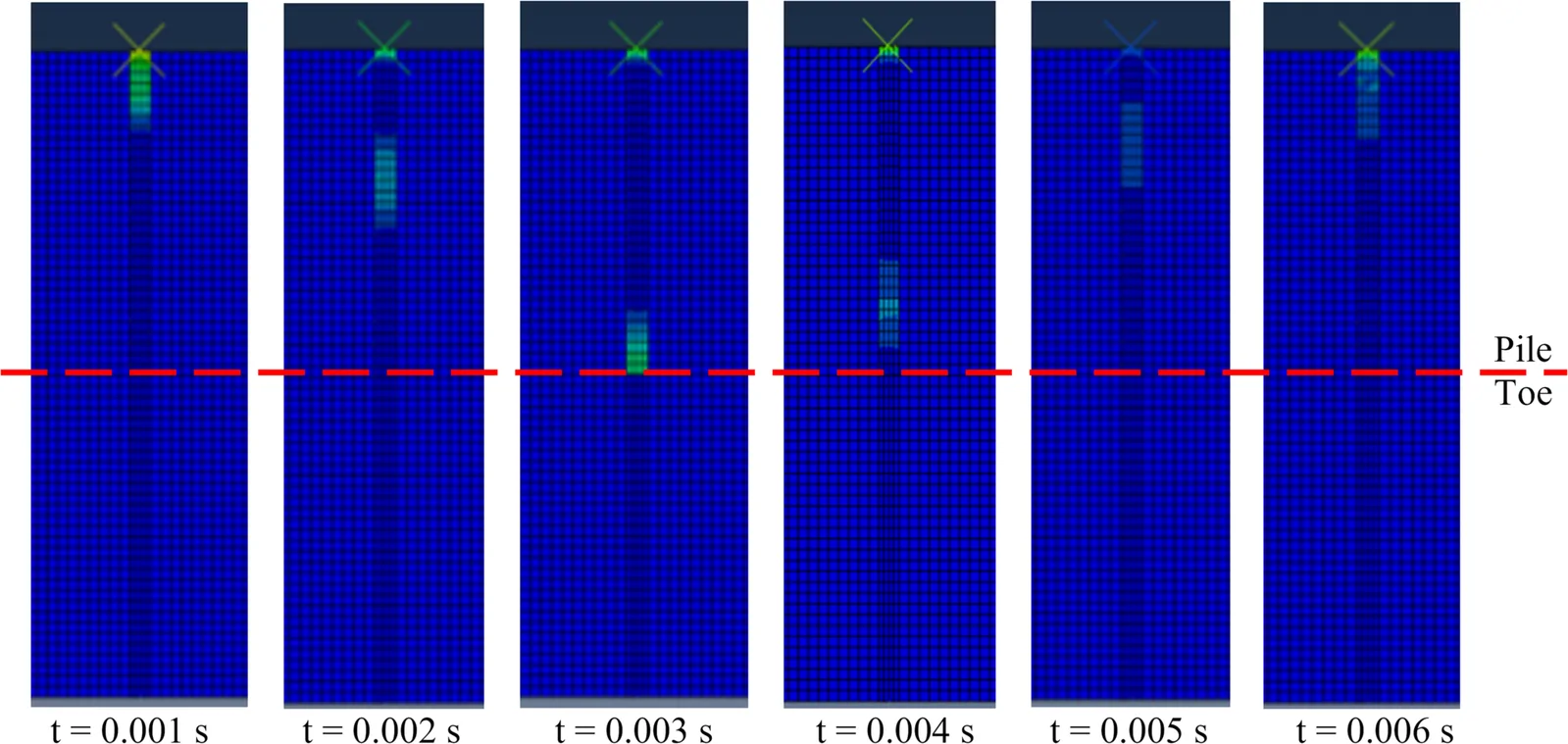
Wave propagation through the pile.

The contour plots of wave propagation in the intact pile are represented in Fig. 9 as it travels along the pile and is reflected. At t = 0.001 s, at the pile head, initiation of a downward traveling compression wave is generated after hammer impact. At t = 0.002 s, the wave has propagated along the pile, moving deeper. At t = 0.003 s, the wave approaches the pile toe, then it starts to travel back. At t = 0.004 s and t = 0.005 s, the upward wave is already reflected and continues to propagate, representing the reflection behavior in an intact pile. At t = 0.006 s, the wave returns and reaches the pile surface, illustrating the low-strain integrity testing.

## Results

**Fig. 10:**
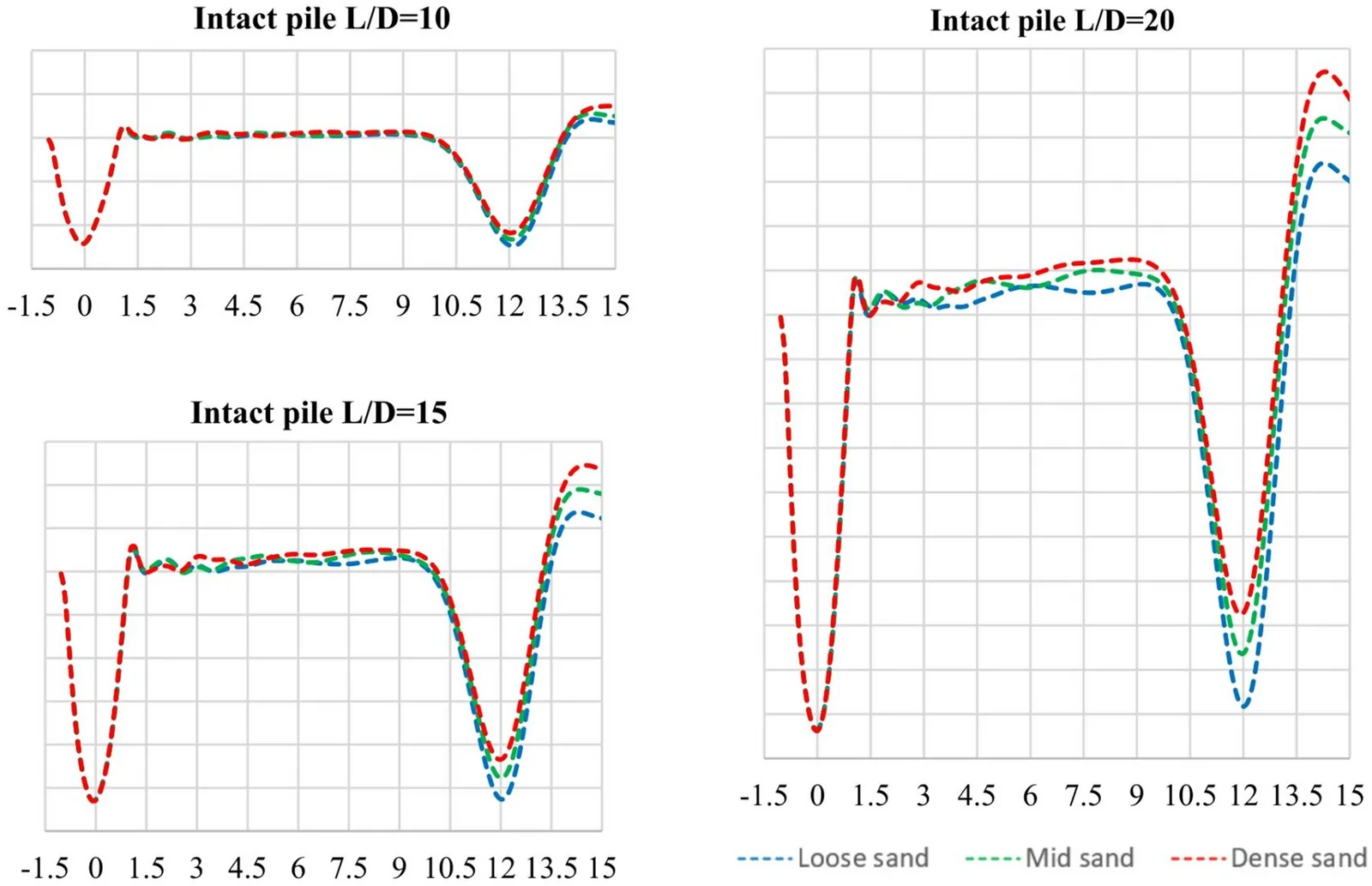
Results (reflectograms) of the intact piles.

The results of the simulated LSIT across various pile slenderness ratios (L/D) and defect types at different soil densities show wave propagation behavior. A key finding across all cases is that the incident wave amplitude decreases as the pile diameter and axial stiffness increase with the same pile length. The resulting reflectograms were presented on a unified vertical scale. For the intact piles, the magnitude of the initial incident peak recorded at the pile head becomes noticeably larger as the L/D ratio increases from 10 to 20, as shown in Fig. 10. Moreover, the reflectogram amplitude at the pile toe is greater for soil 1 than for soil 3. This behavior is related to the larger stiffness contrast between the concrete pile and soil 1. Since the difference between the modulus of elasticity of soil 1 and that of the concrete pile is greater than the corresponding difference for soil 3. This results in a higher amplitude of the reflected wave due to a stronger mismatch at the bottom pile-soil interface.

The results of the simulated Low-Strain Integrity Testing (LSIT) across various pile slenderness ratios (L/D) and defect types at different soil densities illustrate distinct wave propagation behavior. A key finding across all cases is that the amplitude of the incident wave decreases as the pile diameter and axial stiffness increase, while the pile length remains the same.

The reflectogram amplitude of the defect signal decreases significantly with depth. This indicates that energy loss occurs as the wave travels deeper into the pile.

**Fig. 11:**
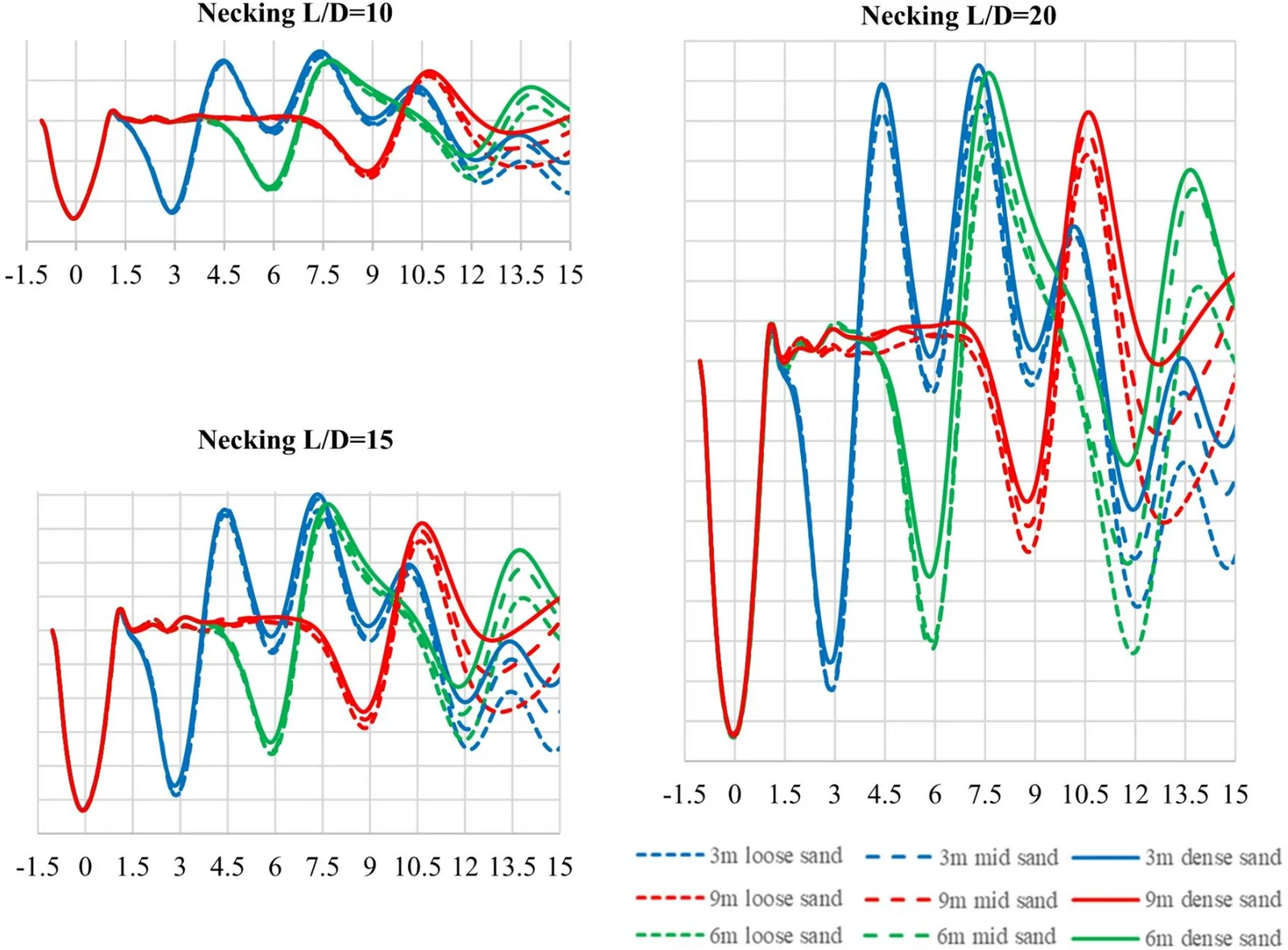
Results (reflectograms) of piles with necking.

**Fig. 12:**
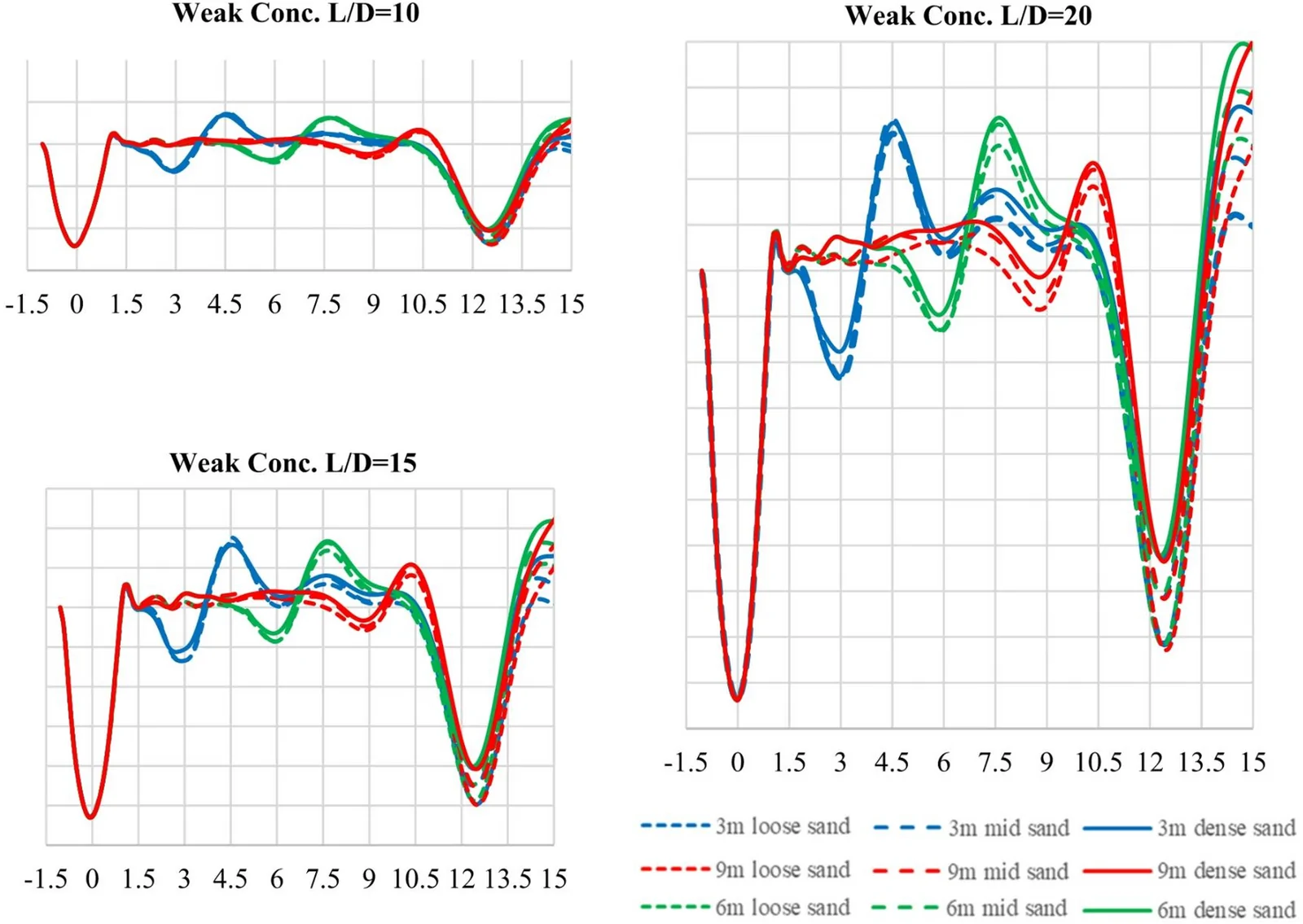
Results (reflectograms) of piles with weak concrete.

**Fig. 13:**
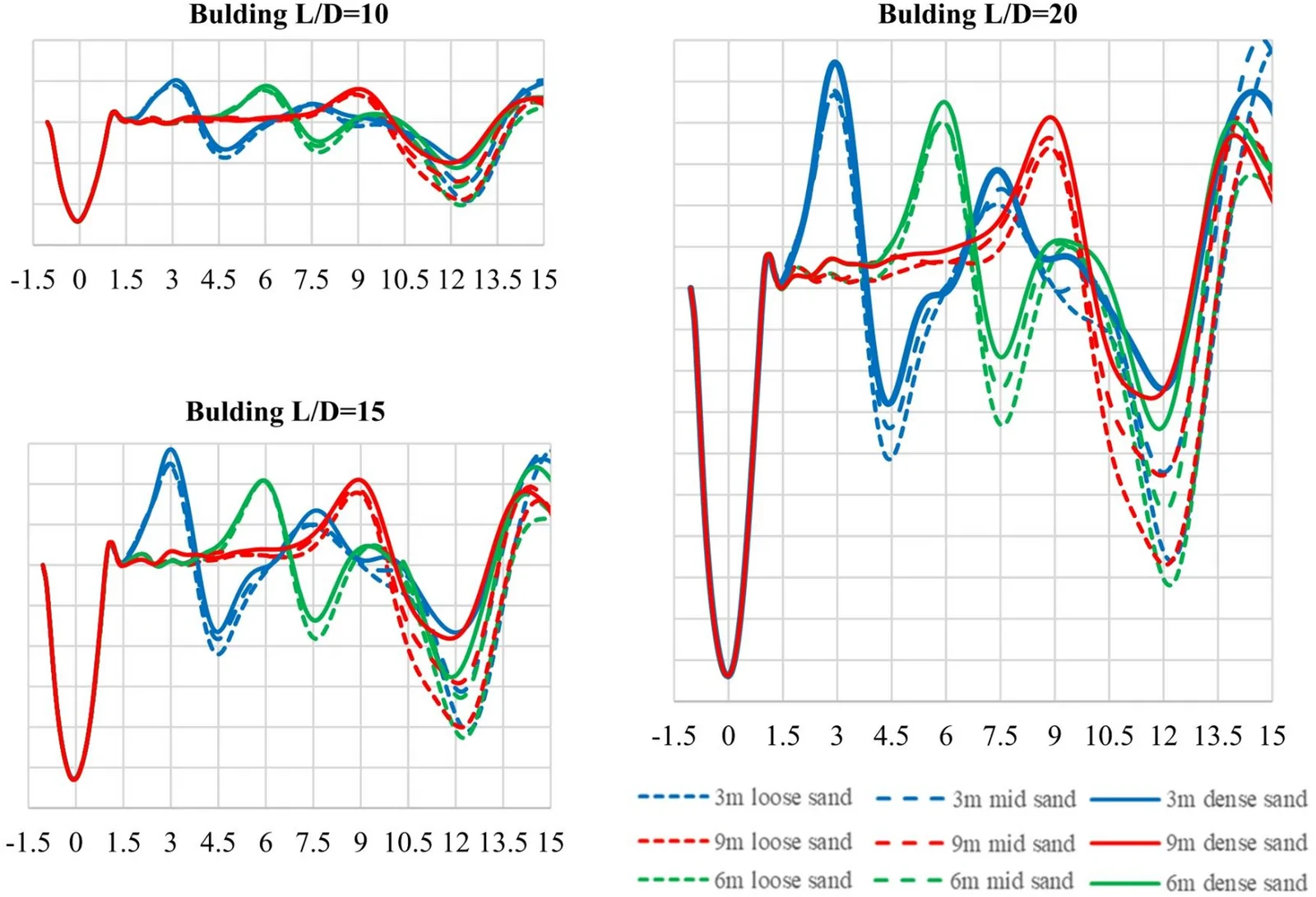
Results (reflectograms) of piles with bulging.

- At the necking location, the signal shows a very clear negative peak followed by a positive one due to the loss of cross-section, as shown in Fig. 11.
- At the weak-concrete zone, the waveform is also negative, then positive, but both peaks are much smaller than for necking. Modulus reduction generates a weaker impedance contrast than area reduction during necking, as shown in Fig. 12.
- At the bulging location, the response reverses this pattern, with a positive peak followed by a negative peak, consistent with the local increase in pile impedance. When the defect is near the toe, the travel-time difference between the defect reflection and the pile toe is very small. This means the two reflected waves overlap in the time record rather than appearing as two separate peaks, as shown in Fig. 13 (Table 3).

**Table 3:**
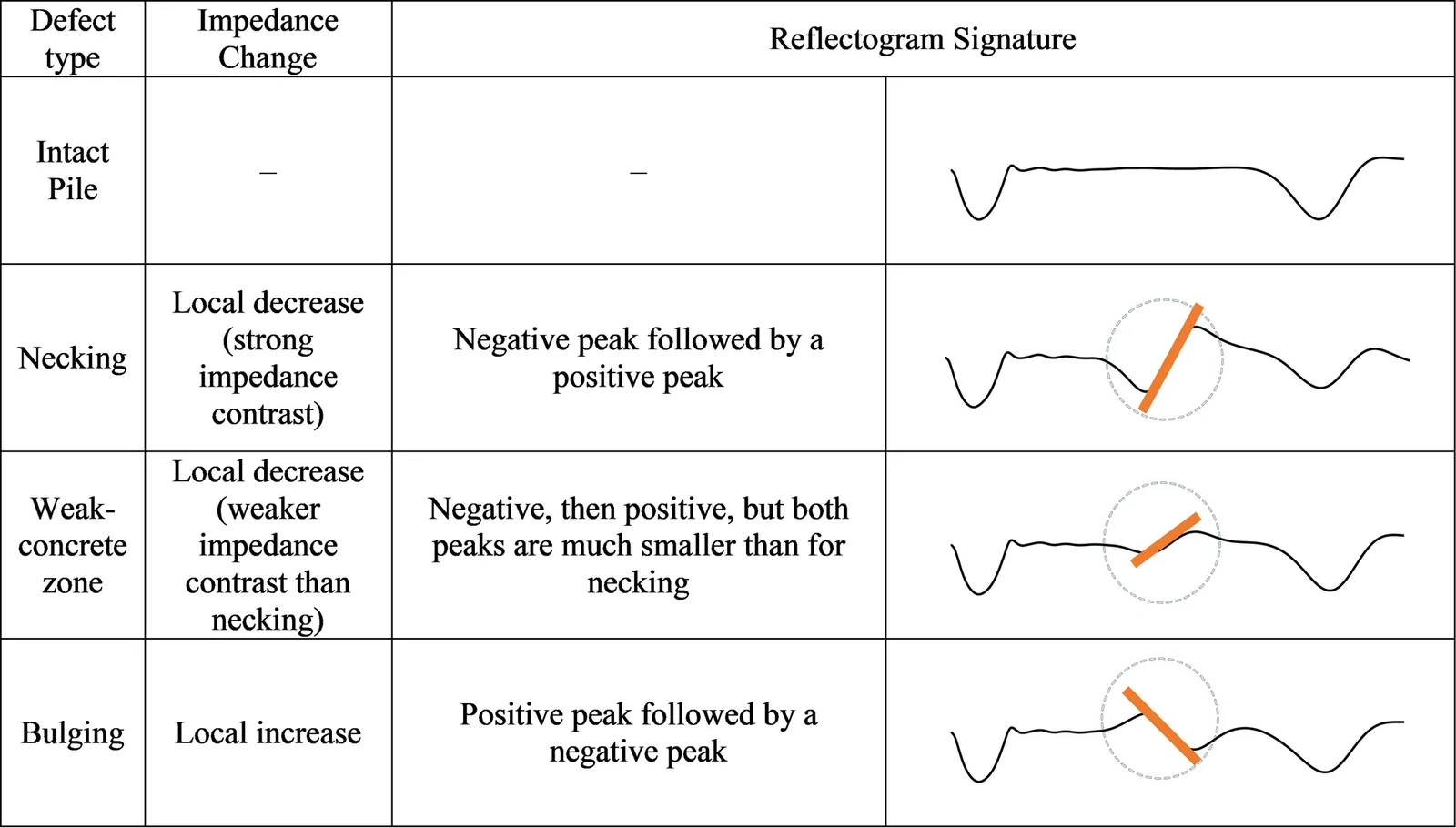
Summary of impedance changes for each defect type.

The delay between the incident peak and the reflected wave at the pile toe depends on the defect location. However, the defect type introduces stress-wave reflection behavior in terms of time-of-arrival delay and waveform shape.

- In necking, the apparent pile length, based on the total wave arrival time, exceeds the actual pile length, as shown by the reflectogram in Fig. 11. The combination of the reflected wave that travels from the pile tip to the defect depth and reflected back to the pile tip, then back to the pile toe, with the original wave shifted to the detected pile toe, causing the depth to exceed the expected pile length. Since the defect reflection arrives first and the toe reflection follows, the combined signal extends over a time that corresponds to the sum of the defect depth and the full pile length. This makes the apparent travel path longer than the actual pile length.
- Weak concrete shows a lower wave speed in that zone. This low velocity results from the reduced pile stiffness. Figure 12 shows a small additional delay in the expected arrival time at the pile toe.

- In the bulging case, it is observed that there is a time delay. It is determined by an increase in the length of the travel path relative to that expected pile length. This happens as the reflections between the enlarged section and the pile toe increase the total signal duration. The interaction of multiple wave reflections along the pile causes the observed signal. The results show that the stress wave generated at the pile head travels downward and is partially reflected from the defect. This reflected wave then propagates toward the pile toe. After undergoing another reflection, it travels back upward. These successive reflections interact with the original wave at the pile surface. Figure 13 represents these wave paths and time shifts. The overlapping negative peak and the reflected wave at the pile toe occur when the defect is located at 75% of the pile length.

## Discussions

**Fig. 14:**
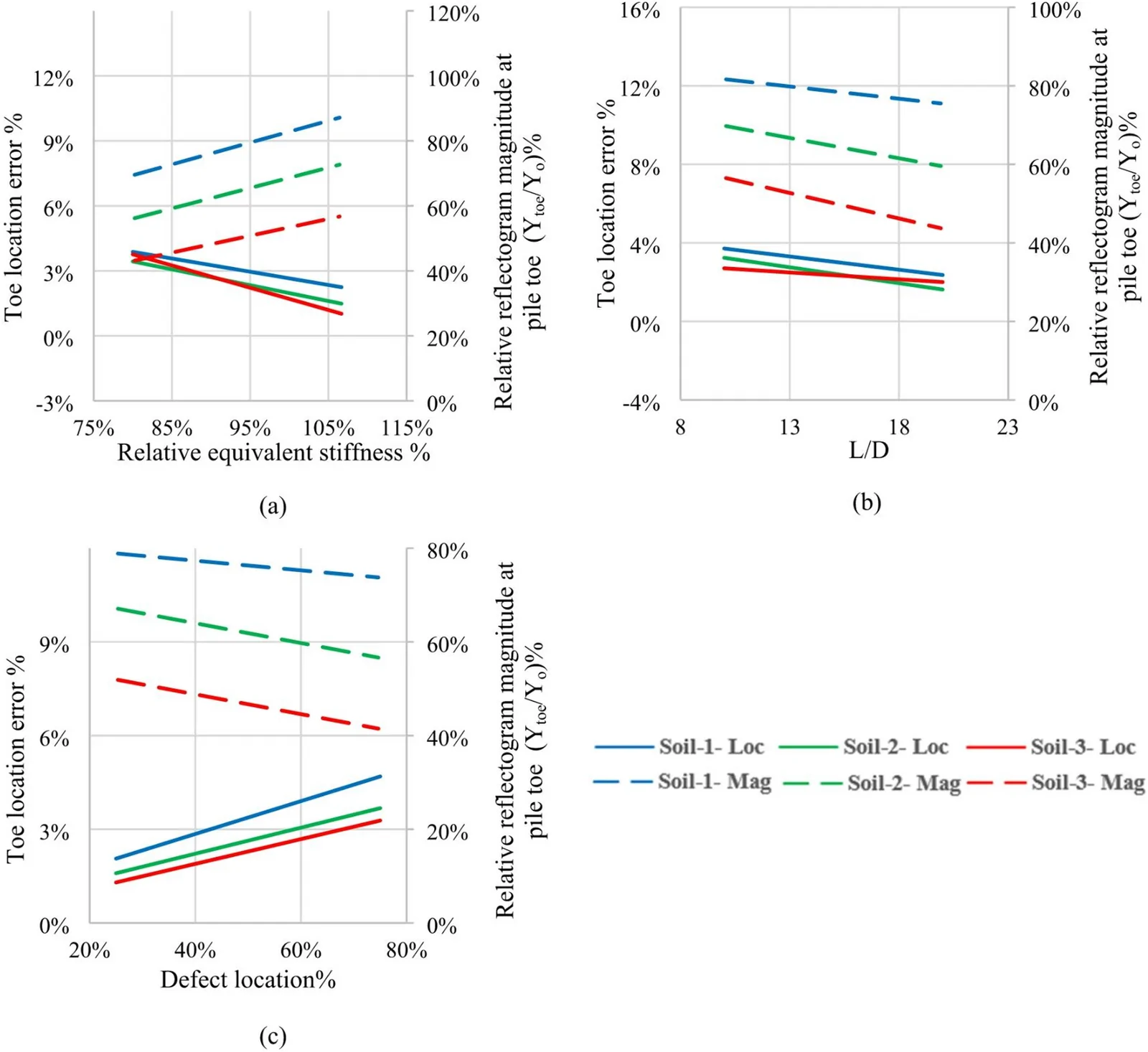
Effect of (**a**) relative stiffness, (**b**) L/D, and (**c**) defect location on toe detection accuracy and relative reflectogram magnitude at the toe in different soil types.

The effects of relative equivalent stiffness, pile slenderness ratio L/D, and defect location are illustrated. These effects are discussed on the toe location error and the reflectogram magnitude at the pile toe. Figure 14 represents these effects, which vary with soil densities. Soil 1 characterizes the loose sand, soil 2 represents mid, and soil 3 is dense sand. Plots of the effects of pile properties and surrounding soil stiffness are shown. These plots highlight their effects on pile toe detection accuracy in LSIT and the relative magnitude reflectogram at the pile toe and defect.

### Effect of relative equivalent stiffness

The effect of relative equivalent stiffness on the pile toe detection is shown in Fig. 14 (a). The toe location error decreases as the relative equivalent stiffness increases for the three sands. This increase in equivalent stiffness is determined from approximately 80% to 107%. These percentages indicate the necking, weak concrete, and bulging defects. For soil 1, the toe location error decreases from about 3.6% to approximately 2.3%. The error decreases from around 3.2% to about 1.6% in soil 2. While in soil 3, the reduction drops from approximately 3.8% to nearly 1.2%. The results indicate that the toe-detection accuracy of the wave signal reduced with increasing stiffness as the wave propagates with less energy loss.

In contrast, the magnitude of the relative reflectogram (Y_toe_ /Y_0_) increases with increasing stiffness. For soil 1, the magnitude increases from about 70% to nearly 85%. Soil 2 shows an increase from approximately 55% to about 74%, while soil 3 increases from roughly 42% to about 58%. The results indicate that higher equivalent stiffness, in which elastic deformation is more than permanent deformation, enhances wave transmission efficiency along the pile, leading to stronger reflections at the pile toe.

### Effect of pile slenderness ratio (L/D)

In Fig. 14 (b), it is observed that as the pile diameter decreases (higher L/D), the toe location error decreases. As the pile diameter increases, the incident wave magnitude (Y_0_) decreases. This behavior can be related to increases in pile impedance and to three-dimensional (3D) wave propagation effects. For L/D = 10, due to the 3D effect, the length of the vertical wave traveling below the 3D effect is less compared to the pile with L/D = 20, resulting in a high toe location error.

Also, the reflectogram magnitude at the toe decreases with L/D because the impedance decreases, which allows more wave energy to be dissipated in the soil, making (Y_toe_ /Y_0_) decrease with L/D. Regarding the 3D effect, which is larger at L/D = 10 than at L/D = 20, and focusing on the vertical (axial) component, the energy dissipated along the pile below the 3D effect zone is lower in the large-diameter pile than in the small-diameter pile, resulting in a higher rebound magnitude at the pile toe. The magnitude of this effect also varies with the surrounding soil type. In soil 1, the relative toe magnitude remains the highest among the three cases, decreasing slightly from about 81% to 76% as L/D increases. In soil 2, moderate soil stiffness leads to increased energy dissipation compared with soil 1, reducing the relative toe magnitude from approximately 69% to 60%. In soil 3, the relative toe magnitude is the lowest, decreasing from about 57% to 42%, reflecting the effect of the interaction between the pile and the surrounding stiff soil.

### Effect of defect location

The effect of defect location is opposite to that of stiffness and L/D with respect to the error in pile toe location. It is shown that, for the three sands, the toe location error increases as the defect position deepens. The toe location error increases from approximately 2% to 4.7% in soil 1. In soil 2, the toe location error increases from 1.5% to 3.6%. For soil 3, the range is from 1.3% to 3.1%. This indicates that deeper defect reflections interfere with the reflection from the pile toe, increasing the error in toe location. On the other hand, the relative toe magnitude at the pile toe (Y_toe_ /Y_0_) decreases with increasing defect depth, indicating greater wave energy dissipation into the surrounding soil due to the longer travel path. The magnitude decreases from about 79% to 74% in soil 1, from 66% to 58% in soil 2, and from 52% to 41% in soil 3, as shown in Fig. 14 (c).

### Effect of defect type

**Fig. 15:**
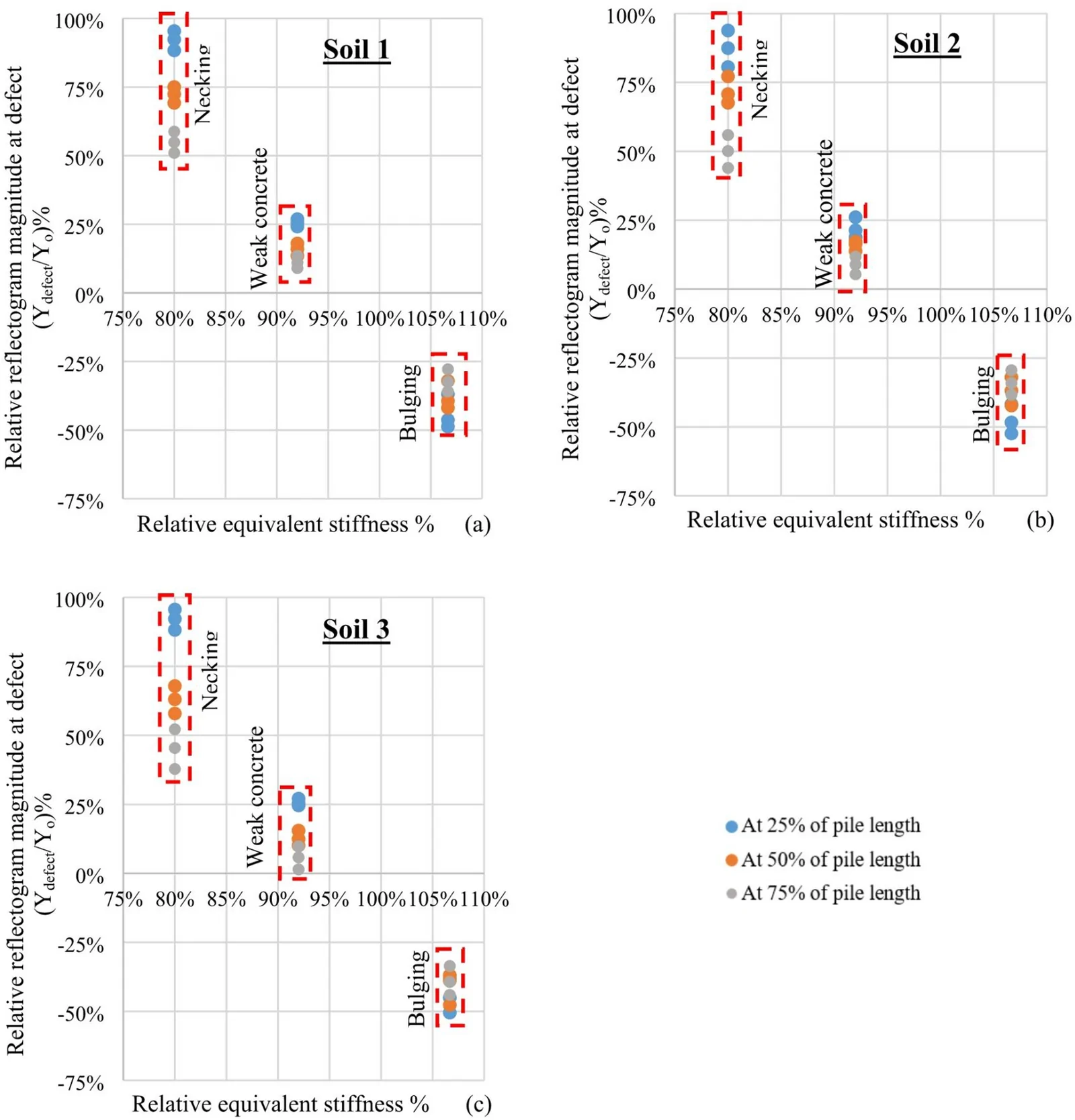
Effect of defect type and soil type on wave reflectogram and relative equivalent stiffness at different (**a**) Soil 1, (**b**) Soil 2, (**c**) Soil 3.

Figure 15 shows that each defect type, necking, weak concrete, and bulging, falls into its own zone when relative equivalent stiffness (K_defect_/K_intact_) is plotted against relative reflectogram magnitude at the defect location (Y_defect_/Y_0_). This enables the assessment of the effect of the relative equivalent stiffness on the magnitude of the reflectogram at the defect location.

- Necking points group is approximately 80% of the intact pile stiffness in soil 1, and their relative reflectogram magnitude at defect values range from roughly 50% to almost 100%. In soil 2, the relative magnitude for shallow defects (25% depth) remains high, reaching nearly 95%, but as the defect moves to 75% depth, the magnitude drops to approximately 45%. In soil 3, while the stiffness stays consistent at 80%, the relative magnitude at 75% depth drops further to nearly 37%. For necking defects, the relative magnitude at the defect location systematically decreases as the defect is located deeper along the pile. At a necking depth of 75%, the reflectogram magnitude is noticeably lower than that of a shallow defect, indicating that the reflected wave becomes weaker as the defect is located deeper in the pile.
- Weak concrete defects reduce the equivalent stiffness only slightly in soil 1, to 92%. Defects at 25% of the pile length result in reflections near 27%, whereas those at 75% depth produce magnitudes as low as 10%. In soil 2, the reflectogram magnitude at the defect ranges from 5% to 25%. In soil 3, the reflectogram magnitude at the defect reaches 25% for shallow defects and nearly 0% for those at 75%. The wave becomes increasingly difficult to interpret at greater depths. This happens especially in cases of low material contrast and low magnitude, due to energy loss at greater depths. This suggests that in soil 3, deep within weak concrete, it is at the highest risk of being undetectable due to extremely low signal contrast.
- In soil 1, bulging defects showed a relative stiffness of nearly 107%, while their relative magnitude ranged from approximately − 50% to − 28%. In soil 2, the relative magnitude ranges from − 29% to -52%. In soil 3, the reflectogram magnitude at the defect ranges from − 34% to -48%. This representation shows that in necking, there is a large drop in impedance compared to the change of impedance for the bulging case, making the relative reflectogram magnitude in necking stronger than in the other defect types.

**Fig. 16:**
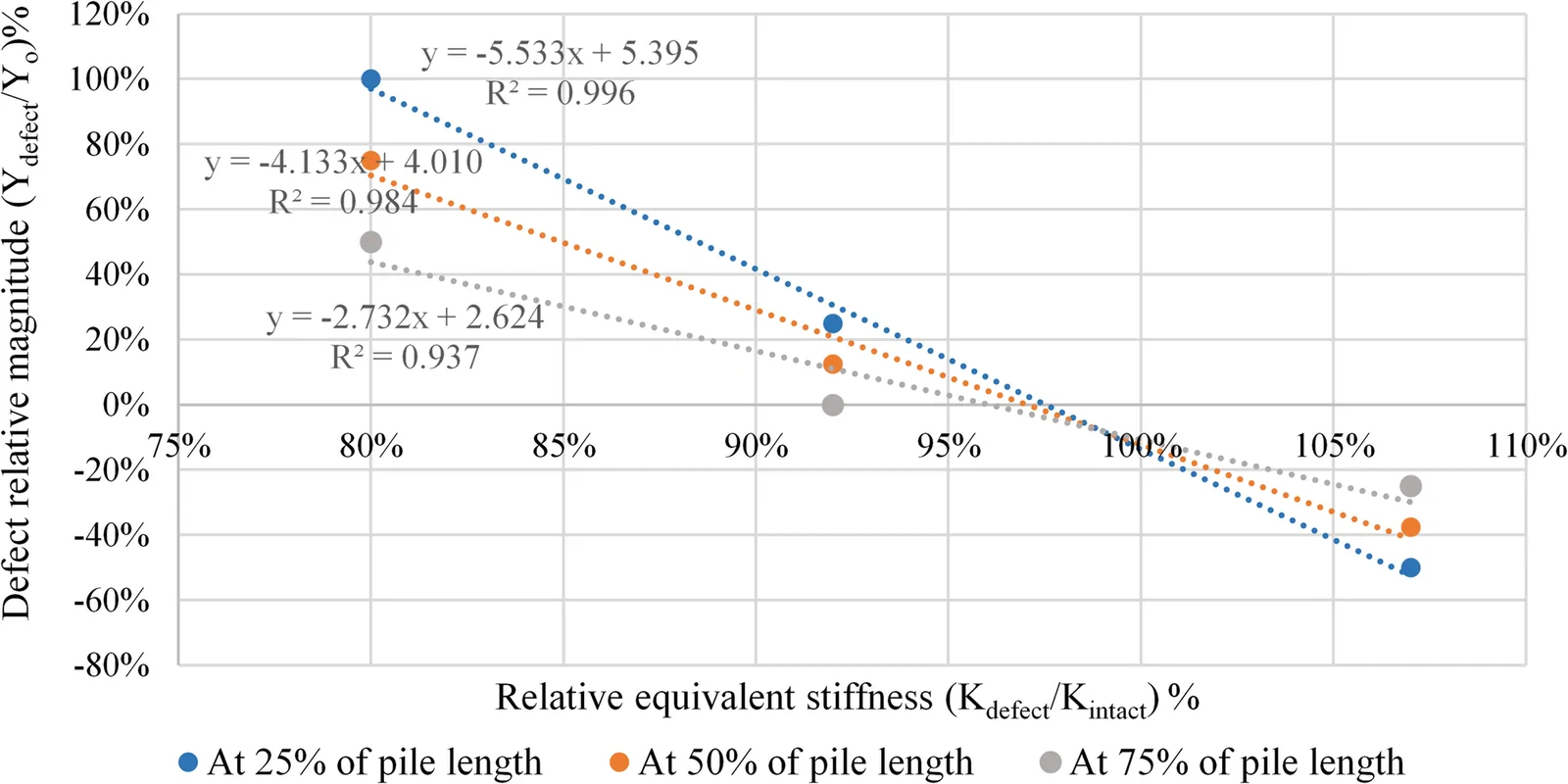
Relationship between defect relative magnitude and relative equivalent stiffness at different defect locations.

The relationship between the relative equivalent stiffness (K_defect_/K_intact_) and the relative defect magnitude (Y_defect_/Y_0_) shows a clear inverse trend across all defect locations investigated (25%, 50%, and 75% of the pile length). In general, as the relative stiffness increases, the relative magnitude of the defect response decreases. This behavior indicates that as the stiffness reduction decreases, the defect’s reflected wave amplitude diminishes correspondingly. Moreover, the rate of decrease in relative magnitude increased as the defect location deepened along the pile. This is illustrated by the steeper slope observed for defects located at 75% of the pile length compared with those at 25% and 50%. The linear regression relationships obtained for each defect location provide a consistent approximation of this trend and confirm the relationship between relative stiffness and defect magnitude (Fig. 16).

**Fig. 17:**
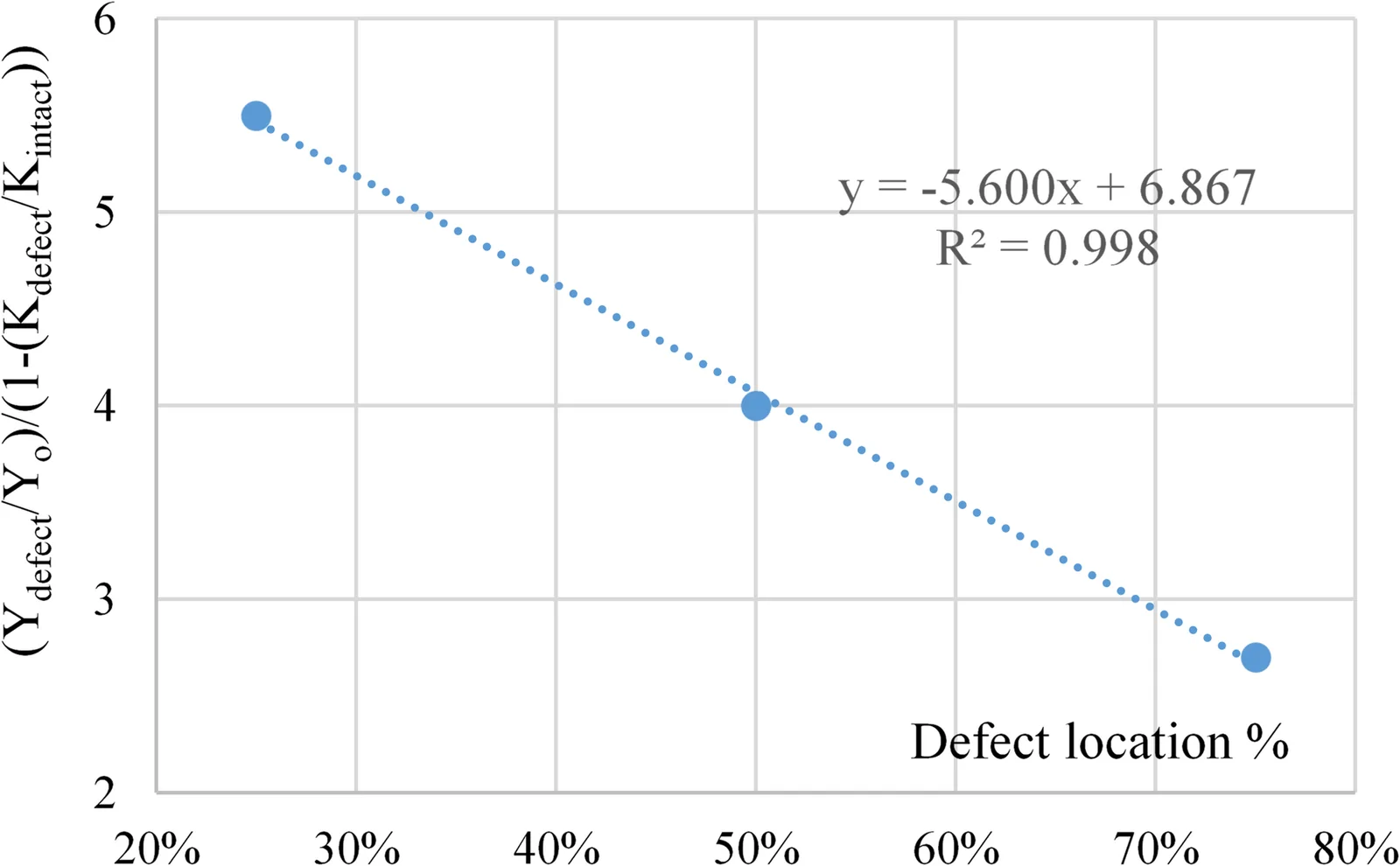
Effect of defect location on the relationship between defect magnitude and relative stiffness.

The relationship between the normalized defect response coefficient (Y_defect_/Y_0_)/(1-(K_defect_/K_intact_)) and the defect location along the pile is shown in Fig. 17. A strong linear relationship is studied, with the coefficient decreasing as the defect location moves deeper along the pile length. Theoretically, when the relative stiffness (K_defect_/K_intact_) approaches 100%, the defect magnitude (Y_defect_ /Y_0_) approaches zero because the pile condition becomes identical to that of an intact pile. However, the plotted values do not reach an exact zero because of the use of averaged results, the linear regression to represent the data, and the approximation introduced when adjusting the relationships in Fig. 16. Based on this relationship, the defect relative magnitude can be expressed as shown in Eq. (10), which provides a practical expression that links the defect response magnitude with both the relative stiffness reduction and the defect location along the pile.

10 $$ \frac{{{\mathrm{Y}}_{{{\mathrm{defect}}}} }}{{{\mathrm{Y}}_{0} }} = \left( {1 - \frac{{{\mathrm{K}}_{{{\mathrm{defect}}}} }}{{{\mathrm{K}}_{{{\mathrm{intact}}}} }}} \right)\left( {6.8667 - 5.6\left( {{\mathrm{defect}}\:{\mathrm{location}}\:{\mathrm{ratio}}} \right)} \right) $$

## Conclusion

This research fulfilled an important gap study regarding the low-strain integrity testing (LSIT) which is the impact of pile defects on the prediction accuracy of pile toe. Accordingly, a parametric study was conducted using 3D FEM model to evaluate the effect of pile slenderness ratio (L/D), defect location, and defect type in different sand densities. A 3D model in ABAQUS 2024 was validated to ensure reliable simulation of wave propagation behavior. This study focuses on their effects on the error in the toe location and on the magnitude of the reflectogram at the pile toe and defect location. A parametric study was carried out on 90 cases, including intact and defective piles. The research outcomes could be concluded in:

- The FEM validation model indicated excellent fitting between the digitized and FEM reflectograms.
- For each defect, the waveform reflectogram has been observed.
  - Necking: strong negative-positive peaks with a high magnitude of reflectogram at the defect.
  - Weak concrete: same as the necking, but with a much smaller reflectogram magnitude.
  - Bulging: positive-negative peak at the defect location.
  - The effect of defect type on toe location error indicates that for a bulging defect of high relative stiffness, the toe location error reduces as the wave travels with less energy loss. The toe location error is reduced by 36% compared to the necking defect for soil (1) It also reduces by 50% in soil 2 and 68% in soil 3. While the reflectogram magnitude at the pile toe increases by about 21%, 34%, and 38%, respectively.
  - Increasing the pile slenderness ratio with a fixed pile length reduces the error of detection of the pile toe due to the minimal effect of the 3D effect. It causes the error to decrease by 22% in soil 1 and 2, and a very minimal reduction in soil (2) The reflectogram magnitude at the pile toe decreases by 6% in soil 1, 13% in soil 2, and 26% in soil 3.
  - As the defect is located near the pile toe, there is a combined reflection wave of the defect and pile toe. The toe location error increases by approximately 135% in soil 1, 140% in soil 2, and 138% in soil (3) The reflectogram magnitude at the pile toe decreases by about 6%, 12%, and 21%, respectively, due to increased energy dissipation of the longer travel path. This proves that deeper defects along the pile lead to high errors in toe location and delays in arrival time.
  - A linear relationship is approximately identified by 53% reduction in the normalized defect response as defect location shifts from 25% to 75% of pile length.

### Limitation of the study

The outcomes of this research are valid only within the considered parameters (pile diameter 0.6, 0.8, 1.2 m), (bulging defect, weak concrete, necking of 1 m depth), (defect locations 25%, 50%, and 75% of pile length), and (loose, mid, dense sand).

### Future work

It is recommended that future studies expand the ranges of these parameters, including different soil layers, different defect sizes, other defect types such as voids and cracks, longer pile lengths, and variable loading conditions.

## Data Availability

The datasets generated and/or analyzed during the current study are available from the corresponding author upon reasonable request.
